# Combination MEK and mTOR inhibitor therapy is active in models of glioblastoma

**DOI:** 10.1093/noajnl/vdaa138

**Published:** 2020-10-15

**Authors:** Karisa C Schreck, Amy N Allen, Jiawan Wang, Christine A Pratilas

**Affiliations:** 1 Sidney Kimmel Comprehensive Cancer Center at Johns Hopkins, Baltimore, Maryland, USA; 2 Department of Oncology, Johns Hopkins University School of Medicine, Baltimore, Maryland, USA; 3 Division of Pediatric Oncology, Johns Hopkins University School of Medicine, Baltimore, Maryland, USA; 4 Department of Neurology, Johns Hopkins University School of Medicine, Baltimore, Maryland, USA

**Keywords:** glioblastoma, MEK inhibitor, MTOR inhibitor, neurosphere, targeted therapy

## Abstract

**Background:**

RAS effector signaling pathways such as PI3K/mTOR and ERK are frequently dysregulated in glioblastoma. While small molecule targeted therapies against these pathways have appeared promising in preclinical studies, they have been disappointing in clinical trials due to toxicity and de novo and adaptive resistance. To identify predictors of glioblastoma sensitivity to dual pathway inhibition with mTORC1/2 and MEK inhibitors, we tested these agents, alone and in combination, in a cohort of genomically characterized glioblastoma cell lines.

**Methods:**

Seven genomically characterized, patient-derived glioblastoma neurosphere cell lines were evaluated for their sensitivity to the dual mTORC1/2 kinase inhibitor sapanisertib (MLN0128, TAK-228) alone or in combination with the MEK1/2 inhibitor trametinib (GSK1120212), using assessment of proliferation and evaluation of the downstream signaling consequences of these inhibitors.

**Results:**

Sapanisertib inhibited cell growth in neurosphere lines, but induced apoptosis only in a subset of lines, and did not completely inhibit downstream mTOR signaling via ribosomal protein S6 (RPS6). Growth sensitivity to MEK inhibitor monotherapy was observed in a subset of lines defined by loss of NF1, was predicted by an ERK-dependent expression signature, and was associated with effective phospho-RPS6 inhibition. In these lines, combined MEK/mTOR treatment further inhibited growth and induced apoptosis. Combined MEK and mTOR inhibition also led to modest antiproliferative effects in lines with intact NF1 and insensitivity to MEK inhibitor monotherapy.

**Conclusions:**

These data demonstrate that combined MEK/mTOR inhibition is synergistic in glioblastoma cell lines and may be more potent in NF1-deficient glioblastoma.

**Key Points**

- Glioblastoma neurosphere models have active RAS signaling pathways including PI3K-mTOR and ERK.
- Adaptive re-activation of parallel signaling pathways is a limitation of single-agent treatment.
- MEK and mTORC1/2 combination treatment has synergistic effects in glioblastoma neurospheres.

**Importance of the Study**

RAS effector signaling pathways such as PI3K/mTOR and ERK are frequently dysregulated in glioblastoma. Unfortunately, small molecule targeted therapies against these pathways have been disappointing in clinical trials for several reasons, including de novo and adaptive resistance. Here, we demonstrate that treatment with either MEK or mTORC1/2 small molecule kinase inhibitors partially reduces glioblastoma neurosphere growth, but also upregulates signaling through compensatory RAS effector pathways. Combined MEK/mTOR inhibitor treatment overcomes adaptive resistance and further inhibits neurosphere growth, particularly in lines with NF1 loss of function. These findings suggest that combined therapy could be effective for a subset of patients with glioblastoma exhibiting loss of NF1 expression, which occurs in 15–20% of glioblastoma, once optimal dosing of the combination in vivo is established.

RAS signaling dysregulation occurs in the majority of glioblastoma (GBM; 90%) and thus has become a focus of avid investigation and efforts in drug development.^1^ Among RAS effector pathways altered in GBM, aberrations in PI3K/mTOR and ERK signaling predominate, with mutations in receptor tyrosine kinases (72%), *PTEN* (41%), *NF1* (10%), and *BRAF* (2%) most common.^2^ Despite promising preclinical data, clinical trials of targeted therapies against EGFR, mTOR, and PI3K have been disappointing in GBM.^3–5^ Even with demonstrable target inhibition in human subjects, development has been limited by both suboptimal antitumor efficacy and significant toxicity.^6,7^

The clinical lack of efficacy of these drugs to date is likely multifactorial, but contributing causes may include activation of parallel signaling pathways that converge upon conserved substrates, or adaptive and acquired resistance after an initial response. Upregulation of compensatory RAS effector pathways in response to mTOR inhibition occurs through a variety of mechanisms, including the upregulation of ERK or WNT signaling.^8–11^ These adaptive changes lead to sustained activation of key downstream targets such as p70-S6 kinase (p70S6K) or ribosomal protein S6 (RPS6), the activation of which can confer resistance to ERK pathway inhibitors.^12,13^ This observation has led to attempts to combine multiple RAS effector targeted therapies for GBM and other cancers.

Targeting multiple RAS effector pathways with a combination of MEK and mTOR kinase inhibitors (MEKi and mTORi, respectively) is a strategy that has been tested in multiple other cancers in early-phase clinical trials.^14–16^ In GBM models, the combination can promote differentiation, inhibit clonogenic growth, and decrease in vivo tumor formation through enhanced inhibition of critical downstream targets like p70S6K.^13^ The antitumor effect of combination therapy appears most prominent in preclinical models of glioma with BRAF mutations or KRAS mutations, though these mutations are relatively rare in adult GBM.^17–19^ Similarly, the efficacy of MEKi/mTORi therapy has been demonstrated in vitro in pediatric low-grade glioma (pLGG) and in vivo in pLGG models with BRAF V600E mutations.^20^ Unfortunately, clinical development has been hampered by significant toxicity without notable clinical benefit in early-phase clinical trials.^14–16^

Identification of a biomarker-defined subset of GBM with increased susceptibility to combined RAS effector signaling blockade might increase the opportunity for the clinical efficacy of these drugs. We speculated whether the loss of NF1 could be such a genomic candidate to predict susceptibility to this combination. *NF1* encodes the GTPase-activating protein (GAP) that facilitates the switching of GTP to GDP moieties in RAS and other targets. Germline loss of the *NF1* tumor suppressor gene underlies the molecular basis of neurofibromatosis type I (NF1), which is characterized by peripheral and central nervous system tumors including GBM. *NF1* somatic mutations are also identified in approximately 15% of sporadic GBM, with homozygous deletion in approximately 3% and loss of NF1 expression through ubiquitination and proteasomal degradation in another 10–15%.^1,21^ Loss of *NF1* and *TP53* together is sufficient to induce high-grade glioma formation in mice.^22^ Moreover, loss of NF1 expression is associated with sensitivity to MEKi monotherapy in a subset of GBM cell lines, though this was not borne out in a clinical trial.^23,24^

We hypothesized that dysregulation of multiple downstream RAS effector pathways drives treatment resistance in GBM through co-activation of mTOR and ERK signaling, and that dual inhibition of these pathways would be effective in a subset of genetically defined GBM. We tested the combination of the FDA-approved MEKi (trametinib, Novartis) and a mTORC1/2 kinase inhibitor currently in clinical trials for GBM (sapanisertib, TAK-228, Takeda) in a panel of genomically characterized GBM tumor lines, in order to identify a mutation profile that might be particularly susceptible to combined therapy.

## Methods

### Cell Lines

HSR-GBM1 (a gift from the Vescovi laboratory) and JHH-GBM10 and JHH-GBM14 (derived at Johns Hopkins University) were cultured from human GBM tissue and maintained as neurosphere cultures in serum-free neurosphere media as previously described.^25,26^ JHU-0879, JHH-136, JHH-520, and JHU-1016B were a gift from the Gregory Riggins laboratory and were maintained as neurosphere cultures in serum-free media.^27^ SkMel-103 and SkMel-113 were a gift from Dr. David Solit, Memorial Sloan Kettering Cancer Center. DBTRG-5MG was a gift from Dr. Jean Mulcahy Levy, University of Colorado, Denver. HEK293T cells were purchased from the American Type Culture Collection. All cells were maintained in appropriate media supplemented with 10% fetal bovine serum. Genomic mutations were identified by targeted sequencing (HSR-GBM1) or next-generation sequencing as previously described (JHU-0879, JHH-520, JHH-136, JHU-1016b).^26,27^ For JH-GBM10 and JH-GBM14, we performed whole-exome sequencing and identified mutations in genes of interest. *O*^6^-methylguanine-DNA methyltransferase status is methylated for HSR-GBM1, unmethylated for JH-GBM10, and otherwise unknown. Cell line identity was verified using short tandem repeat profiling analysis and all cell lines tested negative for mycoplasma contamination except for JHU-1016B, which was treated with Plasmocin (Invivogen) and then retested negative.

### Immunoblotting

Cells were disrupted on ice in 1% NP40 lysis buffer or NETN buffer (Bio-Rad) as previously described.^28^ Protein concentration was determined with Pierce BCA Protein Assay Kit (Thermo Fisher Scientific). Equal amounts of protein were separated by SDS-PAGE, transferred to nitrocellulose membranes, immunoblotted with specific primary and secondary antibodies, and detected by chemiluminescence with the ECL detection reagents, Immobilon Western HRP substrate Luminol Reagent (Millipore) and Luminol Enhancer Solution (Thermo Fisher Scientific). The membranes were imaged on the ChemiDoc Touch Imaging System (Bio-Rad). Representative blots of 3–4 independent experiments are shown. Relative changes in p-RPS6 were quantitated by densitometry analysis using Image J as a function of concentration or treatment time and averaged across all blots.

### Reagents

Antibodies against total ERK, phospho-ERK^thr202/tyr204^, total MEK, phospho-MEK^ser217/221^, phospho-p70-S6 kinase^thr389^, phospho-RPS6^ser235/236^, cleaved PARP, B-actin, phospho-4EBP1^thr37/46^, total AKT, phospho-AKT^ser473^, and phospho-AKT^thr308^ were obtained from Cell Signaling Technology, NF1 (A300-140A) from Bethyl, and Cyclin D1 from Santa Cruz Biotechnology. Trametinib and sapanisertib were purchased from SelleckChem. Drugs for in vitro studies were dissolved in DMSO to yield 10 mM or 1 mM stock solutions and stored at −20°C.

### Growth Assay

Cells were plated in triplicate per condition in 96-well plates, treated immediately with drugs as described, and incubated under standard conditions. Cell growth was quantitated using the Cell Counting Kit-8 (Dojindo) and read using an Epoch microplate spectrophotometer (BioTek). Relative survival in the presence of drug was normalized to untreated controls after background subtraction. For all experiments, at least 3 independent replicates were performed. Synergy was calculated via the Chou-Talalay method using Compusyn (www.combosyn.com) as described previously.^29^ In brief, each dosing experiment was completed at least 3 times, and the effect at each concentration was the mean of those replicates relative to untreated control replicates. The combination index (CI) was calculated where CI less than 1 indicates synergy, CI greater than 1 indicates antagonism, and CI equal to 1 indicates additive effect.

### Real-Time PCR

RNA was extracted using the RNeasy kit (Qiagen) with on-column DNase treatment (Qiagen) according to the manufacturer’s instructions. Reverse transcription was performed using (iScript cDNA Synthesis Kit, Bio-Rad), and qPCR was done using iQ SYBR Green Supermix (Bio-Rad) on a CFX96 Real-Time PCR System (Bio-Rad) according to the manufacturer’s instructions. Three independent experiments (with biological triplicate) were completed for each condition in each cell line. Primers for the following genes were obtained from published literature: *SPRED1*, *CCND1*, *SPRY2*, *DUSP6*, and *GAPDH*.^30,31^ Values were normalized to the housekeeping gene *GAPDH* using the ∆∆CT method.

### Statistical Analysis

Graphing, IC_50_ calculations, and statistical analysis were performed using GraphPad Prism, version 8. ANOVA or multiple *t* tests with Bonferroni correction for multiple comparisons were used to evaluate the difference between conditions. Significant differences are indicated on the figure panel for each condition.

## Results

### GBM Neurospheres Express RAS Effector Pathway Activity

**Figure 1.**
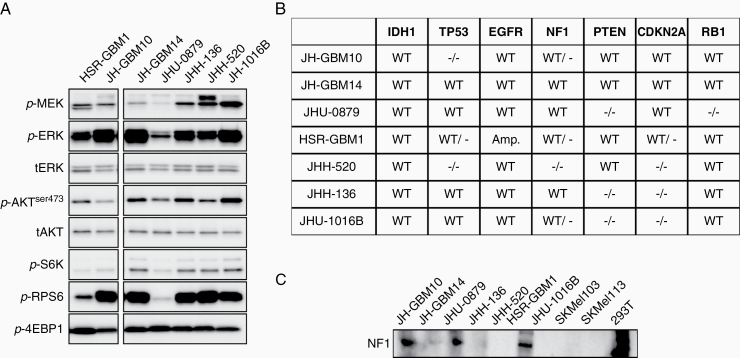
Characterization of a panel of GBM neurosphere cell lines. (A) Immunoblot of 7 neurosphere lines, demonstrating relative expression of ERK and PI3K signaling pathway components. (B) Table summarizing common mutations seen in GBM, in the neurosphere lines used in these experiments. (C) Expression of NF1 protein in the neurosphere lines, 2 cell lines with known loss of NF1 (SKMel-103, SKMel-113, negative controls), and one known to express NF1 (293T, positive control).

In order to assess levels of steady-state pathway activation in the GBM neurosphere cell lines, we evaluated baseline phosphorylation levels of ERK and mTOR RAS effector pathway nodes in cell lines grown in serum-free conditions. Levels of ERK signaling activation were similar between lines, with the exception of JHU-0879, which has *c-myc* amplification (Figure 1A and data not shown). Using published data as well as WES from JH-GBM10 and JH-GBM14, we assembled data on common oncogenic mutations in the panel of cell lines (Figure 1B). All cell lines were *IDH* wild-type. One is *EGFR* amplified (HSR-GBM1), 1 has loss of RB1 (JHU-0879), and 3 have loss-of-function mutations in *TP53* (JH-GBM10, HSR-GBM1, and JHH-520). *NF1* is mutated in one allele in 3 cell lines (JH-GBM10, HSR-GBM1, and JHU-1016B) and in both alleles in JHH-520. Protein expression of NF1 was absent in 3 neurosphere lines (JHH-136, JHH-520, and JHU-1016B), including one with wild-type *NF1* on sequencing (JHH-136; Figure 1C).

### Sapanisertib Causes Growth Inhibition In Vitro But Does Not Completely Suppress mTOR Signaling

**Figure 2.**
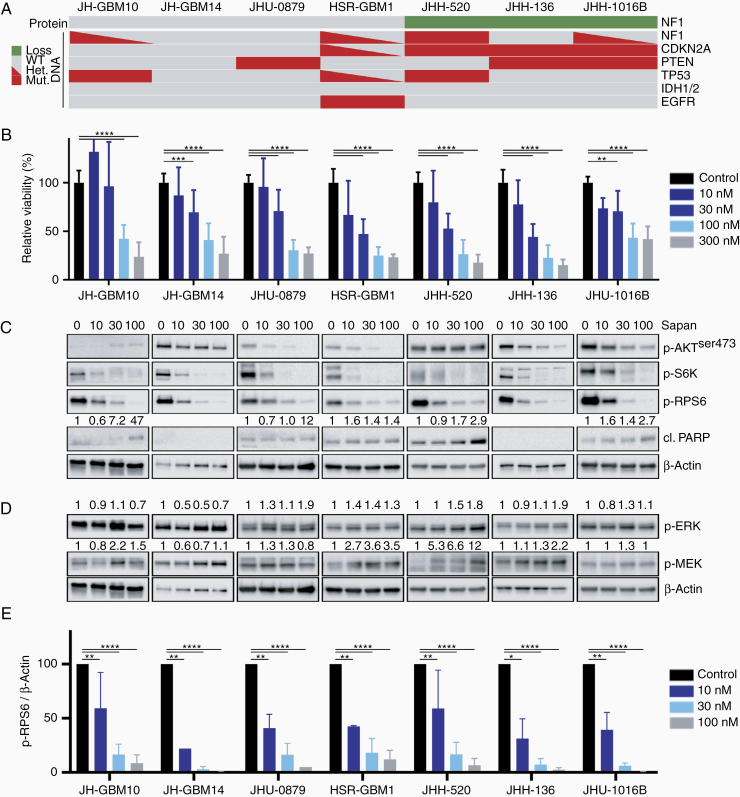
Treatment of glioblastoma lines with sapanisertib. (A) Oncoprint summarizing genomic mutations in *NF1*, *CDKN2A*, *PTEN*, *TP53*, *IDH1/2*, and *EGFR*, and changes in NF1 protein expression in the 7 neurosphere lines. (B) Neurosphere cells were plated in 96-well plates and dosed with increasing concentrations of sapanisertib administered at Day 0. Cell viability relative to untreated control was measured after 96 hours of treatment. (C and D) Immunoblot of AKT and ERK signaling nodes in glioblastoma neurosphere lines after 24 hours of treatment with sapanisertib. Quantification of cl. PARP, p-ERK, and p-MEK relative to β-actin listed above appropriate blot. (E) Quantification of phospho-RPS6 relative to β-actin as measured by quantitative detection on immunoblot, average of 3–4 independent experiments. *P < .02, **P < .005, ***P < .0005, ****P < .00001.

Given the established dependence of GBM on PI3K/mTOR signaling, we treated neurosphere lines with sapanasertib (TAK-228), a dual mTOR1/2 kinase inhibitor, to determine sensitivity to pathway inhibition and antiproliferative effects. Cell growth was inhibited by sapanisertib in a dose-dependent fashion in all neurosphere lines, regardless of genotype (Figure 2A and B). The IC50 for all lines was within a factor of 10, suggesting similar sensitivity to treatment, with a median IC50 of 46 nM (range 16–101 nM; Supplemental Figure 1A). Consistent with its mechanism of action, mTORC1 (readout p-S6K) and mTORC2 (readout p-AKT^ser473^) activity was inhibited in a dose-dependent fashion (Figure 2C). Interestingly, while the phosphorylation of the downstream target RPS6 was similarly inhibited in a dose-dependent fashion across neurosphere lines, inhibition was near-complete in some lines (JH-GBM14, JHH-136, and JHU-1016B) and less complete in others (Figure 2E). Evidence of apoptosis induction was observed in a subset of the cell lines, but did not accurately correlate with the extent of pathway inhibition as detected by immunoblot (Figure 2C). We hypothesized that inhibition of mTOR might relieve negative feedback on other RAS effector pathways such as ERK, leading to a putative mechanism of adaptive resistance. In most lines tested, ERK and/or MEK phosphorylation increased in response to mTORC1/2 inhibition with sapanisertib (Figure 2D). This compensatory effect suggested that ERK signaling activation may be an escape mechanism for treatment with single-agent mTOR inhibitors.

### Trametinib Inhibits Growth in GBM Neurospheres With Loss of NF1

**Figure 3.**
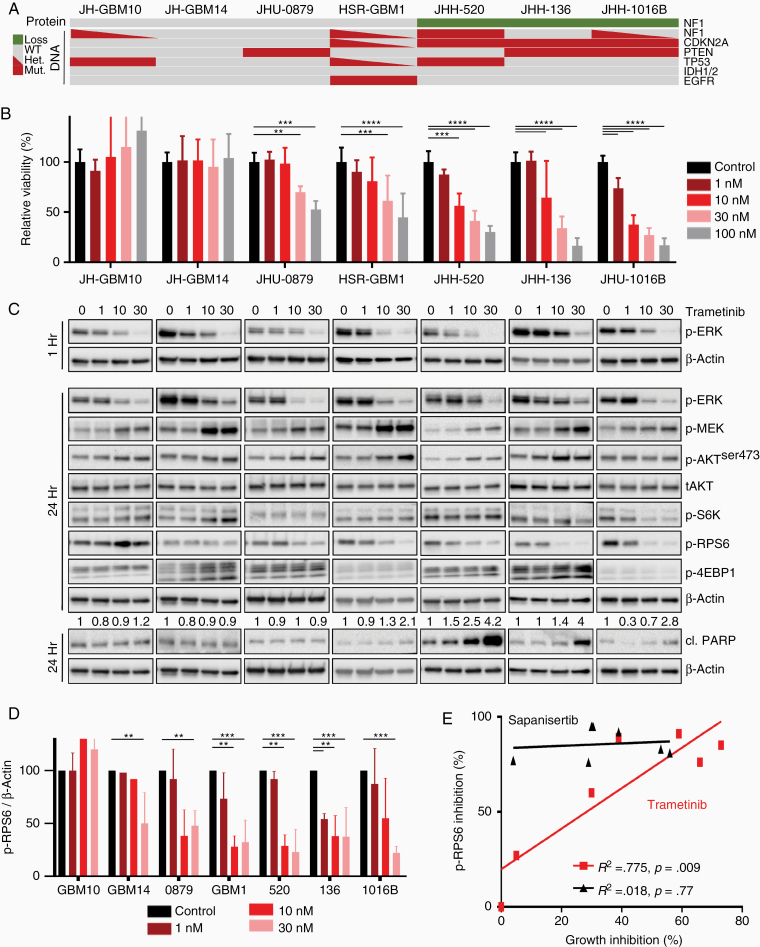
Treatment of glioblastoma lines with trametinib. (A) Oncoprint summarizing genomic mutations in *NF1*, *CDKN2A*, *PTEN*, *TP53*, *IDH1/2*, and *EGFR*, and changes in NF1 protein expression in the 7 neurosphere lines. (B) Neurosphere cells were plated in 96-well plates and dosed with increasing concentrations of trametinib administered on Day 0. Cell viability relative to untreated control was measured after 96 hours of treatment. (C) Immunoblot of AKT and ERK signaling nodes in glioblastoma neurosphere lines after 1 or 24 hours of treatment with trametinib, with cl. PARP quantification relative to β-actin shown above the appropriate band. (D) Quantification of phospho-RPS6 relative to β-actin as measured by quantitative detection of immunoblots, average of 3–4 independent experiments. (E) Growth inhibition in response to trametinib (red, 30 nM) or sapanisertib (black, 30 nM) monotherapy, relative to control cells, was plotted against quantitative phospho-RPS6 inhibition measured by immunoblot, for each cell line. *P < .02, **P < .005, ***P < .0005, ****P < .00001

We therefore investigated whether neurosphere lines might be sensitive to single-agent inhibition of ERK signaling with a small molecule allosteric inhibitor of MEK1/2.^32^ Neurosphere cultures were treated with trametinib and were variably sensitive (Figure 3A and B). Some lines exhibited no growth inhibition in response to trametinib monotherapy (JH-GBM10 and JH-GBM14), while others were quite sensitive (JHH-520 and JHU1016B) and still others had intermediate sensitivity (JHU-0879, HSR-GBM1; Supplemental Figure 1B). Trametinib inhibited ERK phosphorylation in all cell lines at 1 hour but some cell lines began to recover by 24 hours, consistent with prior studies.^30,33^ The downstream target p-RPS6 was only inhibited in those cell lines sensitive to trametinib (Figure 3C).

Apoptosis in response to trametinib was assessed by detection of cleaved PARP, and occurred in a subset of cell lines that also demonstrated growth inhibition (JHH-136, JHH-520, and JHU-1016B), suggesting a higher degree of ERK dependence in those cells (Figure 3C, bottom panel). Notably, the GBM lines most sensitive to MEK inhibition all exhibited loss of NF1 expression, while lines with intact NF1 displayed a lesser, but a variable degree of growth inhibition (Figure 3A and B). This association is in agreement with reports in melanoma, as well as with a previous observation that NF1 loss is associated with MEK inhibitor sensitivity in approximately 30% of high-serum, adherent GBM cultures.^23,34^ Based on these data, we concluded that loss of NF1 expression confers ERK pathway dependence in GBM neurosphere cell lines.

### Growth Insensitivity to Trametinib Is Associated With Increased mTOR Signaling

In order to determine whether mTOR signaling was altered in response to MEK inhibition, we measured levels of phosphorylated AKT, RPS6, and 4EBP1. We observed a dose-dependent increase in markers of mTORC1 and mTORC2 signaling after exposure to trametinib for 24 hours (Figure 3C). Specifically, p-AKTser473, p-S6K^thr389^, and p-4EBP1^thr37/46^ increased, while p-RPS6^ser235/236^ remained stable in the 2 cell lines most resistant to trametinib (JH-GBM10 and JH-GBM14). In cell lines with intermediate sensitivity to trametinib (HSR-GBM1 and JHU-0879), p-AKT increased, while p-S6K and p-4EBP1 remained stable, and p-RPS6 decreased in a dose-dependent fashion. This pattern was in contrast with sensitive lines (JH-520, JHH-136, and JHU-1016B), in which p-S6K remained stable or decreased after trametinib, while p-AKTser473 remained stable or increased.

We hypothesized that compensatory cross-regulation of ERK and AKT/mTOR signaling leads to sustained activity of critical downstream signaling targets such as RPS6, thereby allowing GBM cells to proliferate unabated and escape cell death. We quantified RPS6 phosphorylation changes in response to trametinib treatment and observed dose-dependent inhibition that corresponded closely with the degree of growth inhibition (Figure 3D and E, *R*^2^ = 0.775, *P* = .009). Sapanisertib, on the other hand, inhibited RPS6 phosphorylation in all cell lines without any correlation to the degree of growth inhibition (Figure 3E, *R*^2^ = 0.018, *P* = ns). This finding suggests that in GBM neurospheres, RPS6 phosphorylation may predict sensitivity to MEK inhibition, but not to mTOR inhibition.

### GBM Neurospheres Demonstrate a MEK-Dependence Signature

**Figure 4.**
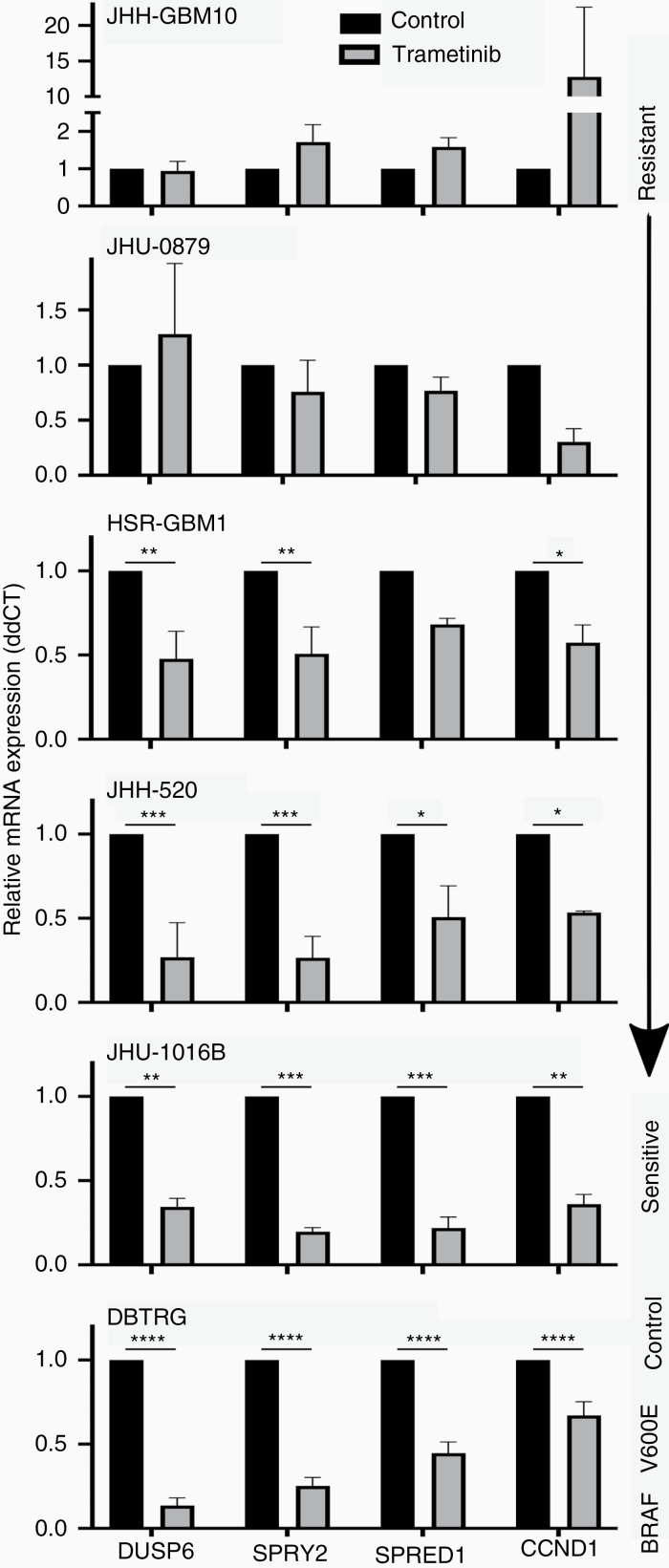
Quantitative PCR of MEK-dependence signature in glioblastoma neurospheres. Four neurosphere lines, representing 1 insensitive to MEKi (JHH-GBM10), 2 with moderate sensitivity (JHU-0879, HSR-GBM1), and 2 with high sensitivity (JHH-520, JHU-1016B), as well as one adherent glioblastoma line with a BRAF V600E mutation and known ERK dependence (DBTRG) were treated with trametinib (30 nM) for 6 hours. mRNA levels for *DUSP6*, *SPRY2*, *SPRED2*, and *CCND1* were measured using quantitative RT-PCR, normalized to the housekeeping gene *GAPDH*. This figure represents 3 independent experiments with technical triplicates. *P < .02, **P < .005, ***P < .0005, ****P < .00001.

Given their differential sensitivity to MEK inhibition, we evaluated whether neurosphere lines exhibited different ERK-dependence signatures. We measured an mRNA expression panel of 4 genes, selected from among those associated with ERK dependence following treatment with trametinib (*DUSP6*, *SPRY2*, *SPRED1*, and *CCND1*).^30^ Expression of these 4 genes was potently suppressed after exposure to trametinib in sensitive lines, whereas in insensitive lines, their expression increased (Figure 4). Other neurosphere lines showed modest inhibition of ERK-dependent targets, consistent with their partial sensitivity. The pattern of sensitivity in MEKi-sensitive lines was similar to what is seen in DBTRG, a GBM cell line with the *BRAF* V600E mutation, which confers ERK dependence and sensitivity to MEK inhibition (Figure 4).^35^

### Combined Therapy With Sapanisertib and Trametinib Is Synergistic in Neurospheres

**Figure 5.**
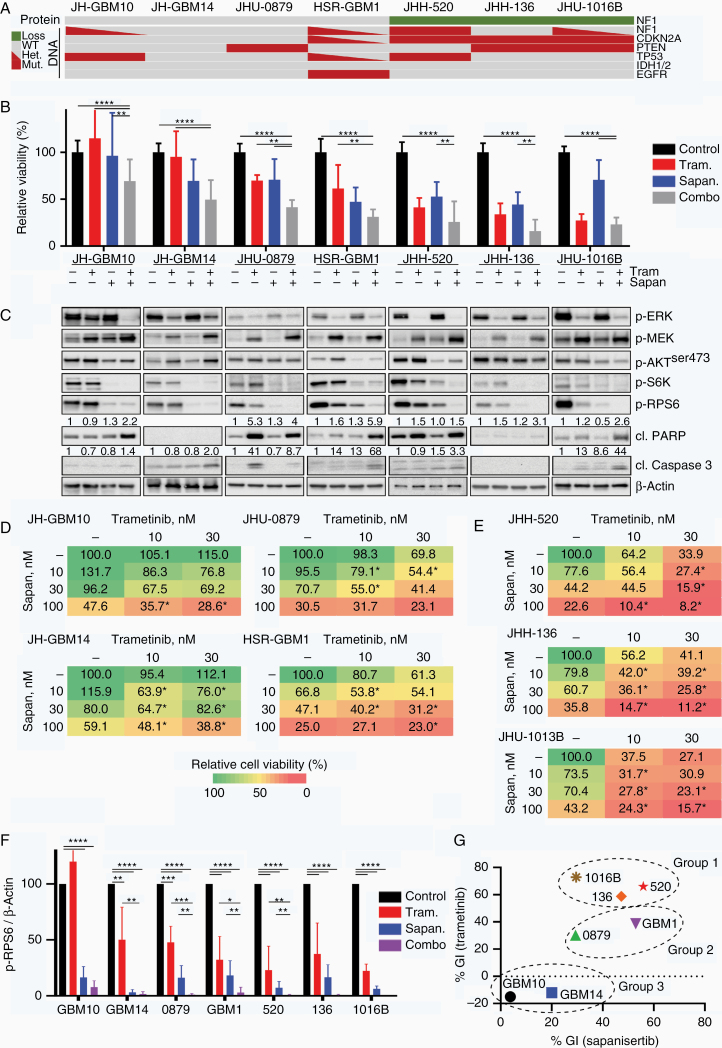
Trametinib and sapanisertib combination is synergistic in neurospheres. (A) Oncoprint summarizing genomic mutations in *NF1*, *CDKN2A*, *PTEN*, *TP53*, *IDH1/2*, and *EGFR*, and changes in NF1 protein expression in the 7 neurosphere lines. (B) Neurosphere cells were plated in 96-well plates and dosed with increasing concentrations of sapanisertib (Sapan, 30 nM), trametinib (Tram, 30 nM), or the combination administered on Day 0. Cell viability relative to untreated control was measured after 96 hours of treatment. (C) Immunoblot of AKT and ERK signaling nodes in glioblastoma neurosphere lines after 24 hours of treatment with sapanisertib and/or trametinib (30 nM each). Quantification of cl. PARP and cl. Caspase 3 relative to β-actin listed above appropriate blot. (D and E) Heatmap of cell viability relative to control at 96 hours, in response to trametinib (10 or 30 nM) and/or sapanisertib (10, 30, or 100 nM) in neurospheres with (D) intact NF1 or (E) absent NF1. * indicates synergistic doses by Chou-Talalay method. (F) Quantification of phospho-RPS6 relative to β-actin as measured by quantitative detection of immunoblots, average of 3–4 independent experiments. (G) Growth inhibition (GI) relative to control in response to trametinib (30 nM) or sapanisertib (30 nM), respectively, for each cell line. Cell lines cluster into 3 groups based on their relative sensitivity. All plotted data are averages of 3–5 independent experiments. *P < .02, **P < .005, ***P < .0005, ****P < .00001.

Our data suggest that RAS-effector pathway conservation may be critical in GBM and occurs via multiple, redundant pathways (such as mTOR and ERK), thereby conferring resistance to single-agent targeted therapy. We tested this hypothesis using combination therapy with selective inhibitors of mTOR (sapanisertib) and MEK (trametinib). We observed dose-dependent growth inhibition with combination therapy in most neurosphere lines (Figure 5A and B). Evidence of apoptosis increased with combined therapy in more than 50% of neurosphere lines (JH-GBM10, HSR-GBM1, JHH-136, and JHU-1016B) and was independent of NF1 expression status (Figure 5C).

We clustered cell lines into 3 groups based on their relative sensitivity to sapanisertib or trametinib monotherapy (Figure 5G). Group 1 was sensitive to trametinib monotherapy and contained all lines with loss of NF1 expression. Group 2 displayed intermediate sensitivity to monotherapy with trametinib or sapanisertib. Group 3 was completely insensitive to trametinib and relatively insensitive to sapanisertib at lower doses. The most significant benefit of combination therapy was evident in Group 1 (Figure 5E). There was also a synergistic effect of combined therapy compared to either drug alone, in Groups 2 and 3, though not as pronounced as in Group 1 (Figure 5D). We calculated synergy between trametinib and sapanisertib using the Chou-Talalay method and observed a combination index demonstrating synergy for all cell lines (Supplementary Figure 2). RPS6 phosphorylation was further inhibited with combination therapy compared to either single drug, in all cell lines (Figure 5F).

## Discussion

The critical role of RAS effector signaling, primarily through the PI3K/mTOR pathway, has been extensively studied in GBM. Unfortunately, resistance to monotherapy with agents against EGFR or other components of the PI3K/mTOR signaling pathway has been well documented.^36^ Several potential mechanisms for resistance have been identified, including upregulation of glutamate metabolism, WNT signaling, or ERK activity.^13,37,38^ In line with these observations, we demonstrated that sapanisertib, an ATP-competitive mTORC1/2 inhibitor currently being evaluated in human clinical trials, was effective at inhibiting growth in neurosphere lines, but only induced apoptosis in a subset of neurospheres and did not completely inhibit downstream targets like p-RPS6. Instead, we observed a dose-dependent increase in ERK pathway activation in most cell lines, likely secondary to loss of feedback inhibition.^7^ Given the inability of this mTORC1/2 inhibitor to completely inhibit downstream signaling, we evaluated dependence on multiple RAS signaling pathways in this system.

MEK inhibitor monotherapy demonstrated marked inhibition of ERK signaling in all GBM neurosphere lines tested in our studies, as expected. Only a subset of lines, however, demonstrated growth inhibition indicative of ERK pathway dependence. Our cell line models could be arranged into 3 clusters of MEKi sensitivity, independent of mTORi sensitivity: low, intermediate, and high. Growth sensitivity also correlated with altered mRNA expression of transcripts known to be associated with a MEK-dependence signature in melanoma and other ERK-dependent tumors.^30,31^ Changes in expression of this signature may be a useful predictive biomarker for clinical responses, but will need to be validated in patient-derived xenograft models and prospective human trials. Notably, the MEK-dependence expression signature did not correlate with sensitivity to combination therapy, suggesting it is truly a readout of ERK dependence alone, not overall RAS dependence. In our studies, all lines sensitive to MEKi monotherapy had loss-of-functional NF1, either due to genomic mutations or by other mechanisms of expression loss. Lines with intact NF1 expression demonstrated intermediate or no sensitivity to MEKi. This finding was consistent with prior studies demonstrating that loss of NF1 may be a predictive biomarker for ERK-dependent regulation of cell proliferation.^34,39^ Combined inhibition of mTOR and MEK pathways led to a synergistic antiproliferative effect in many GBM lines, regardless of NF1 expression. We observed 2 different patterns of synergy: (1) In lines with loss of NF1, mTORi sensitized cells to the effects of MEKi, with enhanced apoptotic and antiproliferative effect. This finding is in agreement with previous data that demonstrated sensitivity in high-serum, adherent GBM lines with loss of NF123. (2) Neurosphere lines that were insensitive to MEKi monotherapy demonstrated synergy to combined MEK/mTOR inhibition, particularly at higher, biologically relevant doses of sapanisertib and trametinib. While the degree of growth inhibition in vitro was not as profound as for lines with NF1 loss, some lines with almost no sensitivity to either drug alone showed demonstrable sensitivity to the combination. These data suggest that combination therapy may produce benefit even for GBM patients without obvious genomic predictors of RAS-effector dependence.

The purpose of this study was to evaluate whether identifiable genomic mutations, specifically loss of *NF1*, in GBM sensitize neurosphere models to targeted therapy with mTORi and MEKi. We hypothesized that loss of NF1 might sensitize cells to combined therapy, given preliminary data suggesting that a MEKi alone can induce growth suppression in some GBM cell lines.^23^ Here, we observed that loss of NF1 demonstrated a moderate correlation with sensitivity to MEKi monotherapy and combination therapy—a powerful observation given 10–15% of GBM has lost functional NF1 expression.^1,21^ Loss of neither the tumor suppressor *PTEN* nor *TP53* correlated with sensitivity to combination therapy. Given the high frequency of RAS signaling alternations in GBM (~90%), it is possible that other mutations or amplifications not identified by our targeted sequencing are present in these GBM lines and contributed to their sensitivity.^2^ Additionally, our findings in this small number of neurosphere lines will need to be validated in a larger cohort.

mTOR kinase inhibitors have produced a range of toxic side effects in patients in whom they have been tested, particularly when combined with MEK inhibitors in Phase I trials. Moving such a combination forward into future clinical trials may be a challenge. We nonetheless selected this particular combination given its relative specificity to explore the biological vulnerabilities of our cell line models, and further work is ongoing to optimize a dosing strategy that will be tolerable for human testing while sustaining biologically significant pathway inhibition. If dosed appropriately and with a tolerable profile of side effects, combination therapy with targeted mTOR and MEK inhibitors could potentially prove efficacious for patients with GBM regardless of genomic background.

## Supplementary Material

vdaa138_suppl_Supplementary_Figures_S1-S2

## Notes

**Authorship statement**. Conception and design: K.C.S. and C.A.P.; development of methodology: K.C.S., A.A., and J.W.; acquisition, analysis, and interpretation of data: K.C.S., A.A., and C.A.P.; writing, review, and revision of the manuscript: K.C.S., A.A., J.W., and C.A.P.
