# Supplementary material for: Combination MEK and mTOR inhibitor therapy is active in models of glioblastoma
Source: Neurooncol Adv. 2020 Oct 15;2(1):vdaa138. doi: 10.1093/noajnl/vdaa138 (PMC7668446; doi:10.1093/noajnl/vdaa138)
Supplement: vdaa138_suppl_Supplementary_Figures_S1-S2 [file vdaa138_suppl_supplementary_figures_s1-s2.pdf]

Supplemental Figure 1

A

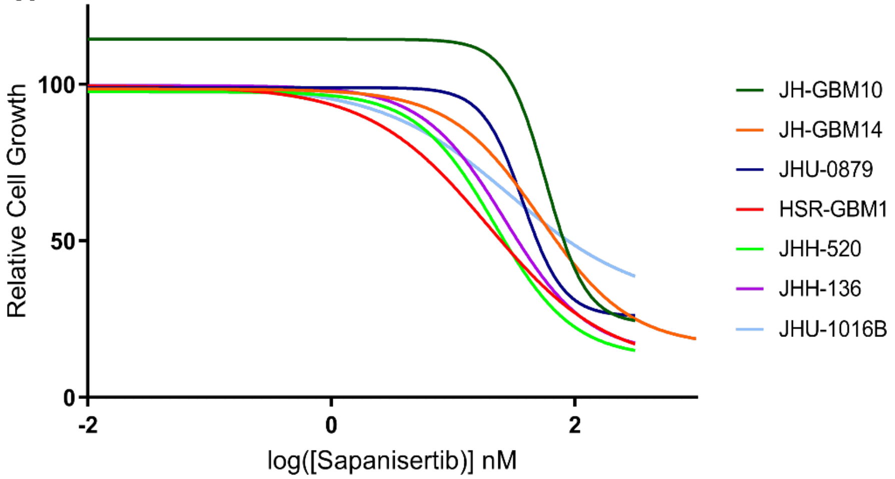

JH-GBM10	70
JH-GBM14	63
JHU-0879	50
HSR-GBM1	17
JHH-520	20
JHH-136	39
JHU-1016B	97

B

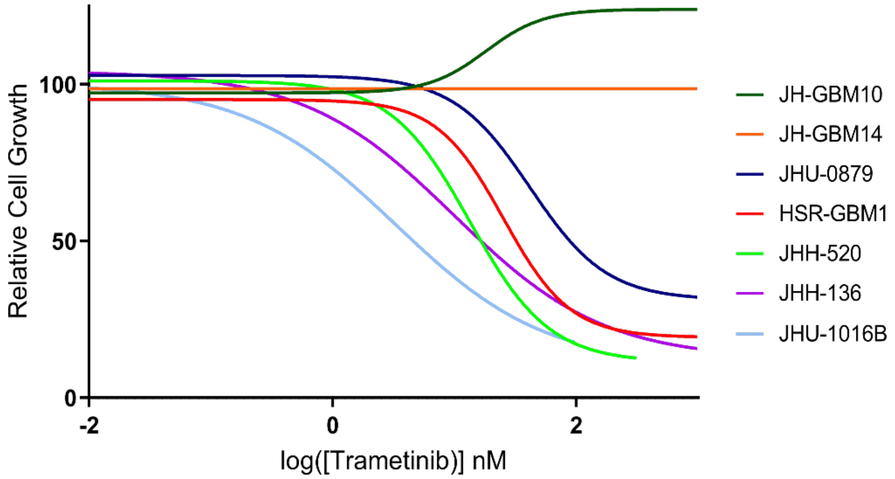

JH-GBM10	>300
JH-GBM14	>300
JHU-0879	25
HSR-GBM1	83
JHH-520	15
JHH-136	19
JHU-1016B	12

Supplemental Figure 1. Mean inhibitory concentrations of single-agent targeted therapy in neurosphere lines. Neurosphere cells were plated in 96-well plates and dosed with increasing concentrations of (A) trametinib or (B) sapanisertib administered at D0. Cell viability relative to time 0 was measured after 96 hours of treatment and dose-response curves were plotted based on growth inhibition. Curve fitting and IC50 values (nM) plotted in GraphPad using a variable slope non-linear inhibitor-response curve fit. All data are averaged over 4-6 independent experiments.

Supplemental Figure 2.

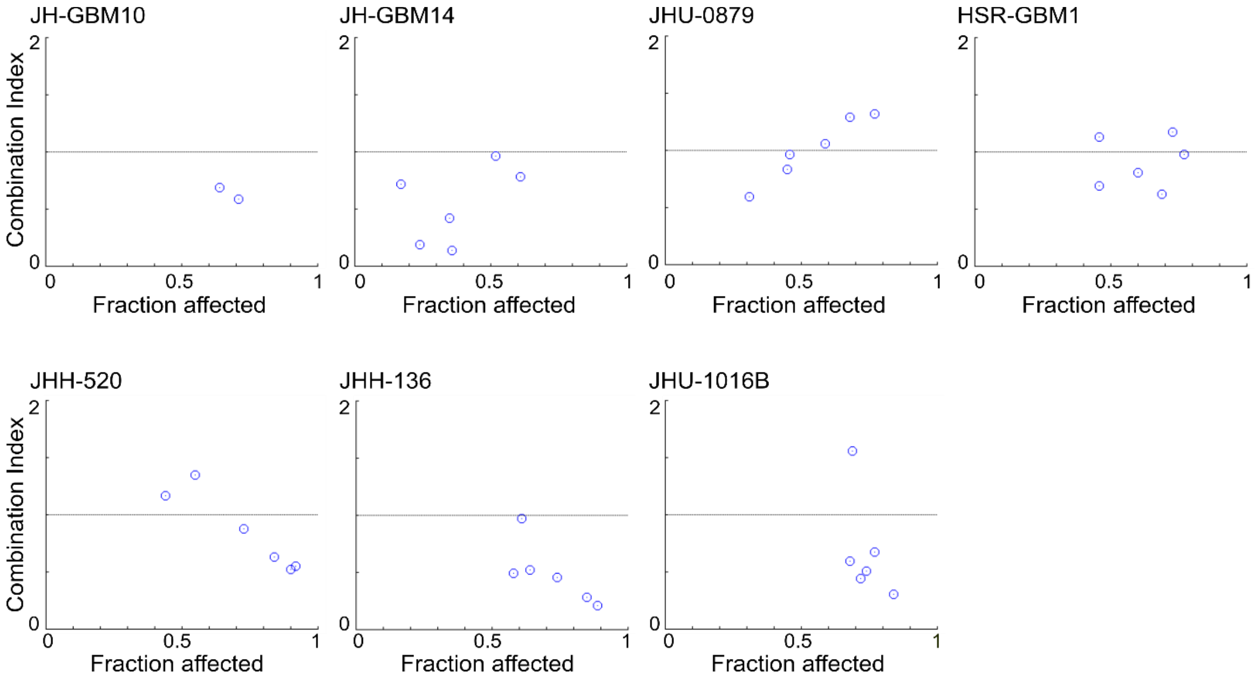

Supplemental Figure 2. Combination indexes for synergy between sapanisertib and trametinib in neurosphere lines. Data obtained from www.combosyn.com using the Chou-Talalay method for a range of paired doses of trametinib and sapanisertib alone and in combination. Doses with CI < 1 are synergic, CI = 1 are additive, and CI > 1 are antagonistic.
